# Barriers to a safety checklist and methods to improve usage of the WHO safety checklist in interventional radiology

**DOI:** 10.1259/bjrcr.20150128

**Published:** 2016-05-02

**Authors:** Thomas Puttick, Archie Speirs, Matthew Gibson, James Tadjkarimi, Farhan Ahmad

**Affiliations:** Department of Radiology, Royal Berkshire NHS Foundation Trust, Reading, UK

## Abstract

Our aims were to first assess uptake of the modified safety checklist (SC) for interventional radiology (IR), identify obstacles to using the SC, and then apply changes to local policy to reach maximum compliance. Retrospective data collection was performed of all patients who underwent an interventional procedure in the interventional suite at the Royal Berkshire Hospital in February, March and May 2014. Barriers to a SC: a lack of awareness about the SC; lack of training in how to complete the SC; lack of leadership—no team member had been given the role of promoting the SC and collecting and collating the SC; attitude of staff—some team members felt that the SCs were time consuming and further bureaucracy; out-of-hours procedures involved staff from outside departments who were not familiar with the SC; paper copies of the completed SCs were being misplaced. Results: February 2014 = 79%; staff education of the importance of the modified World Health Organization (WHO) checklist disseminated in the interventional suite and at clinical governance; each day a designated ‘SC champion’ in the interventional suite has the responsibility for overseeing the WHO checklist is completed for each patient; the use of a clipboard for storing checklists, amalgamated and scored at the end of each day. Any checklists not completed are highlighted and discussed with the consultant. March 2014 = 95%; junior nurse involvement in auditing to improve awareness and engagement; out-of-hours interventional radiologist ensuring that the checklist is completed for each patient. May 2014 = 100%.

## Background

Interventional radiology (IR) is a procedure-based speciality with parallels in surgery. Both can result in significant morbidity with potential for serious complications. As in surgery, IR procedures take place in defined areas, such as the IR suite, where teamwork and, above all, safety requirements are equivalent.^1^ With this in mind, it is appropriate that IR should be subject to the same safety practices and standards as surgery.

In 2008, the World Health Organization (WHO) introduced the surgical checklist to reduce mortality, morbidity and errors in surgical procedures.^2^ Shortly after, Haynes et al^2^ carried out a large multicentre trial to test its efficacy and demonstrated resounding success, proving that simple but effective guidelines could significantly reduce poor outcomes. Following suit, the Royal College of Radiologists assisted with the implementation of a modified WHO checklist specific to the IR.^3,4^ The checklist was further modified for use specifically at the Royal Berkshire Hospital (Appendix A).

In our IR department, compliance with the modified IR WHO checklist was suboptimal, and to improve implementation, we identified barriers to its usage. Our aims were to first assess uptake of the modified safety checklist (SC) for IR, identify obstacles to using the SC, and then apply changes to local policy to reach maximum compliance.

## Materials and methods

Once the proposal was registered with the Trust R&D at the Royal Berkshire Hospital, we collected retrospective data on all patients who underwent an IR procedure in the interventional suite in February, March and May 2014. We then identified perceived barriers to the SC usage in IR by holding focus groups with radiographers, nurses and doctors working in IR. We then implemented changes in an attempt to increase the SC usage. Data was collected for each individual case, including indication, procedure, outcomes and whether a checklist had been fully completed. If there were missing data or a checklist was missing, this was highlighted and collated for each month and discussed at clinical governance meetings.

## Results

Following discussions with consultant radiologists and radiographers (Table 1), we identified the following barriers to SC uptake:

1. A lack of awareness about the SC.
2. Lack of training in how to complete the SC.
3. Lack of leadership—no team member had been given the role of promoting the SC and collecting and collating the SC.
4. Attitude of staff—some team members felt that the SCs were time consuming and further bureaucracy.
5. Out-of-hours procedures involved staff from outside departments who were not familiar with the SC.
6. Paper copies of the completed SCs were being misplaced.

**Table 1.** Use of WHO-modified SC over a period of 3 months

Month of assessment in 2014	Cases using the WHO SC (%)
February	79
March	95
May	100

SC, safety checklist; WHO, World Health Organization.

## Discussion

Barriers to staff adoption of surgical SCs are well documented.^5–7^ The Royal College of Radiologists WHO checklist was further optimized for interventional procedures performed at the Royal Berkshire Hospital to make every question relevant to our clinical practice. For example, the section on general anaesthetic was removed from our standard checklist as very few patients at our trust undergo general anaesthesia for the interventional procedures. On the rare occasions it is necessary the original checklist was used instead.

The SC were either completely filled in or not completed at all, there were no partially completed forms. Although usage of the SC was initially high, there was room for improvement. Our biggest barrier was staff attitude and reluctance to change. This was addressed through education about “never events” and demonstrating checklist usage in surgery. We highlighted the importance of patient safety and made it a priority for IR frontline staff. We made completing the checklist compulsory and made sure that all team members were aware that this was a requirement for every case.

Between February and March 2014, we introduced several specific measures designed to increase usage. We disseminated information in the IR suite and at regular clinical governance meetings. We designated a specific departmental “SC champion” who promoted and facilitated SC usage. Rotating nurse involvement in auditing the checklist helped improve awareness and engagement with its usage. All completed SCs were immediately added to a designated clipboard that was readily available. SCs were collected and scored at the end of the day and any absent checklists were highlighted to the team performing the procedure. For out-of-hours procedures, we made it the responsibility of the IR consultant to complete the checklist. The SC champion informed the consultant if the SCs were not completed or unavailable to ensure that it became routine for out-of-hours cases. By adopting these measures, we achieved 100% compliance.

This case study highlights important learning outcomes in IR. Primarily, 100% compliance with SCs enables team members to voice concerns and should help reduce errors in IR by providing a systematic process to reduce morbidity and mortality. Engaging all members of the IR team, rather than just specific groups ensured effective use of the SC. The IR team felt considerable pride at achieving 100% usage rate, which should help ensure continuation at this level.

## Learning points

1. Increased compliance with the WHO checklist enables team members to voice concerns, which can reduce errors in clinical practice.
2. Involvement of all team members in the audit process in IR helped to achieve 100% compliance.
3. Having a checklist provides a systematic process to reduce morbidity and mortality.

## References

[1] World Alliance for Patient Safety WHO guidelines for safe surgery. Geneva: World Health Organization; 2008 [Accessed 2982015]. Available from: http://www.who.int/patientsafety/safesurgery/tools_resources/SSSL_Checklist_finalJun08.pdf?ua=1.

[2] Haynes AB, Weiser TG, Berry WR, Lipsitz SR, Breizat A-H, Dellinger EP, et al A surgical safety checklist to reduce morbidity and mortality in a global population. N Engl J Med 2009; 360: 491–9. 19144931 10.1056/NEJMsa0810119

[3] Koetser ICJ, de Vries EN, van Delden OM, Smorenburg SM, Boermeester MA, van Lienden KP A checklist to improve patient safety in interventional radiology. Cardiovasc Intervent Radiol 2013; 36: 312–19. 22562482 10.1007/s00270-012-0395-z PMC3595473

[4] Corso R, Vacirca F, Patelli C, Leni D Use of “time-out” checklist in interventional radiology procedures as a tool to enhance patient safety. Radiol Med 2014; 119: 828–34. 24651938 10.1007/s11547-014-0397-9

[5] de Vries EN, Hollmann MW, Smorenburg SM, Gouma DJ, Boermeester MA Development and validation of the SURgical PAtient Safety System (SURPASS) checklist. Qual Saf Health Care 2009; 18: 121–6. 19342526 10.1136/qshc.2008.027524

[6] Fourcade A, Blache J-L, Grenier C, Bourgain J-L, Minvielle E Barriers to staff adoption of a surgical safety checklist. BMJ Quality Safety 2012; 21: 191–7. 10.1136/bmjqs-2011-000094 PMC3285141 22069112

[7] Bergs J, Lambrechts F, Simons P, Vlayen A, Marneffe W, Hellings J, et al Barriers and facilitators related to the implementation of surgical safety checklists: a systematic review of the qualitative evidence. BMJ Qual Saf 2015; 24: 776–86. 10.1136/bmjqs-2015-004021 26199428

